# Realising radical potential: building community power in primary health care through Participatory Action Research

**DOI:** 10.1186/s12939-023-01894-7

**Published:** 2023-05-17

**Authors:** Denny Mabetha, Temitope Ojewola, Maria van der Merwe, Reflect Mabika, Gerhard Goosen, Jerry Sigudla, Jennifer Hove, Sophie Witter, Lucia D’Ambruoso, On behalf in collab the Verbal Autopsy with Participatory Action Research (VAPAR)/Wits/Mpumalanga Department of Health Learning Platform

**Affiliations:** 1 Cochrane South Africa, South African Medical Research Council (MRC), Cape Town, South Africa; 2 MRC/Wits Rural Public Health and Health Transitions Research Unit (Agincourt), School of Public Health, University of the Witwatersrand, Johannesburg, South Africa; 3 Aberdeen Centre for Health Data Science, Institute of Applied Health Sciences, School of Medicine, Medical Sciences and Nutrition, and Centre for Global Development, School of Education, University of Aberdeen, Aberdeen, Scotland, UK; 4 Health Education England, Northwest, Manchester, England, UK; 5 Maria Van Der Merwe Consulting, White River, South Africa; 6 Mpumalanga Department of Health, Mbombela, South Africa; 7 Institute for Global Health and Development, Queen Margaret University, Edinburgh, Scotland, UK; 8 Department of Epidemiology and Global Health, Umeå University, Umeå, Sweden; 9 Public Health, National Health Service (NHS) Grampian, Aberdeen, Scotland, UK; 10 Department of Global Health, Stellenbosch University, Stellenbosch, South Africa

**Keywords:** Community participation, Participatory action research, Power, South Africa

## Abstract

**Background:**

While community participation is an established pro-equity approach in Primary Health Care (PHC), it can take many forms, and the central category of power is under-theorised. The objectives were to (a) conduct theory-informed analysis of community power-building in PHC in a setting of structural deprivation and (b) develop practical guidance to support participation as a sustainable PHC component.

**Methods:**

Stakeholders representing rural communities, government departments and non-governmental organisations engaged through a participatory action research (PAR) process in a rural sub-district in South Africa. Three reiterative cycles of evidence generation, analysis, action, and reflection were progressed. Local health concerns were raised and framed by community stakeholders, who generated new data and evidence with researchers. Dialogue was then initiated between communities and the authorities, with local action plans coproduced, implemented, and monitored. Throughout, efforts were made to shift and share power, and to adapt the process to improve practical, local relevance. We analysed participant and researcher reflections, project documents, and other project data using power-building and power-limiting frameworks.

**Results:**

Co-constructing evidence among community stakeholders in safe spaces for dialogue and cooperative action-learning built collective capabilities. The authorities embraced the platform as a space to safely engage with communities and the process was taken up in the district health system. Responding to COVID-19, the process was collectively re-designed to include a training package for community health workers (CHWs) in rapid PAR. New skills and competencies, new community and facility-based alliances and explicit recognition of CHW roles, value, and contribution at higher levels of the system were reported following the adaptations. The process was subsequently scaled across the sub-district.

**Conclusions:**

Community power-building in rural PHC was multidimensional, non-linear, and deeply relational. Collective mindsets and capabilities for joint action and learning were built through a pragmatic, cooperative, adaptive process, creating spaces where people could produce and use evidence to make decisions. Impacts were seen in demand for implementation outside the study setting. We offer a practice framework to expand community power in PHC: (1) prioritising community capability-building, (2) navigating social and institutional contexts, and (3) developing and sustaining authentic learning spaces.

**Supplementary Information:**

The online version contains supplementary material available at 10.1186/s12939-023-01894-7.

## Background

Over 3 billion people, located largely in low- and middle-income countries (LMICs), lack access to essential health care [1]. Health inequalities are widening and deepening amid a slowing global economic outlook and essential services have been devastated following an estimated 17.2 million deaths from COVID-19 [2, 3]. Health inequalities, the systematic differences in health and wellbeing that are unnecessary and unfair, are driven by social and structural factors over which individuals have little or no control [4–6]. For example, early years malnutrition predisposes to both under and overweight, reinforced through e.g., continued poor diet, low physical activity, substance use, occupational hazards, and poor living conditions [7, 8]. Health inequalities are thus social issues with social causes and can only be solved with collective, social action [9, 10]. Social perspectives provide viewpoints of health inequalities as socially constructed, with social and health systems reflecting, and reinforcing harmful social norms [11, 12]. By extension, health systems, through the ways they are configured and operate, can also confront inequalities. From this position, those most directly affected by health inequalities should be directly involved in action to address them.

The active participation of communities in service planning and delivery has long been recognised as a pro-equity approach in primary health care (PHC). The World Health Organization (WHO) Declaration of PHC centralised community participation [13]. PHC was declared as essential, made universally accessible to communities through their full participation and forming an integral part of the country's health system and overall social and economic development [14].Four decades on, widespread support for participation endures: *“The road to UHC [Universal Health Coverage] … runs through a strong, bold, and unwavering government engagement with communities, especially the most vulnerable. At the heart of that engagement is a participatory space for health that allows for meaningful dialogue and debate and serves to amplify the voices of those to whom the health system belongs – its users.”* [15]. Funders and donors increasingly mandate coproduction and community engagement and involvement (CEI) [16, 17], and nation states are experimenting with participatory democracy and participatory innovations such as citizens assemblies [18–20].

In practice, however, participation takes many forms including different actors in different ways. In some cases, participatory activities can reproduce the very power asymmetries they seek to confront [21]. Arnstein’s classic model of citizen participation organised interpretations on a continuum from passive, empty rituals with communities as recipients (e.g., mass immunisation), to people-centred approaches (e.g., community-controlled processes, identifying and addressing own needs) [22]. The model supports a critical appreciation of participation and locates its true goal as radical transformation and empowerment through community formation and solidarity, and as a political process concerned with democratic power as a response to social injustice [23, 24]. Disparate approaches mean that participation is complex to operationalise and presents problems for policy makers [15]. Recent work suggests that participation is more usefully understood as a process; with community empowerment and health improvements comprised of complex processes influenced by social, political and health systems contexts [25, 26]. Most recently, there is recognition that the central category of power is severely under-theorised [6, 14, 27]. As a result, there is limited operational guidance on *how* participation works, especially in terms of expanding popular agency to address unjust and illegitimate use of power and challenge health inequalities at different levels.

In this paper, we present an analysis of community power-building in PHC through a participatory action research (PAR) programme located in a setting of multiple, structural deprivation in rural South Africa, and how efforts were made to develop the process as a sustainable PHC component. PAR is rooted in an emancipatory enquiry paradigm concerned with democratic power as a response to social injustice [28, 29]. It is a critical enquiry process for social change that recognises knowledge production reflects and reinforces existing power relations [30]. It has been applied widely to understand and address health inequalities [23, 24, 27, 31, 32]. In the following section, we describe the context within which the programme was located and in which we sought to support the development of collective capabilities for transformation of health inequalities through generating, acting on and learning from new knowledge.

Nearly three decades after the brutal apartheid regime, post-apartheid societal progress is deteriorating in South Africa, which in 2022 is the most unequal country on earth [33–35]. Over half the population lives in poverty and unemployment is 34% [36, 37]. A generation after its emergence, HIV prevalence is 40–50 times higher in black versus white populations, with HIV risks eight times higher in adolescent females versus males [38]. Entrenched health and social inequalities notwithstanding, the democratic order was and is committed to inclusive development. In 1994, the health sector was transformed from a system of institutionalised racism into a PHC system focused on equitable provision, prevention, promotion, and participatory governance [39]. There are deep disconnects between policy and practice, however. The less and least-advantaged majority are heavily dependent on an under-resourced public sector characterised by systemic underinvestment, multiple human resource crises, corruption and deteriorating infrastructure [40, 41]. The public system deals with a complex disease burden: chronic comorbidities prevail as people live longer with HIV/AIDS. There were 8.2 million known HIV positive in 2017, 2 million not on treatment (many with TB), skyrocketing obesity and heart disease, and injury and violence mortality double the global average [42–46].

Acknowledging the two-tier health system, National Health Insurance (NHI) was introduced in 2011 as a structural reform and commitment to UHC [47]. PHC Re-engineering is the initial implementation strategy encompassing a major revival of the district health system (DHS) focussed on prevention, health promotion and community involvement [48]. A key pillar of the strategy is Ward-Based PHC Outreach Teams (WBPHCOTs) providing home and community-based services linked to PHC facilities, with community health workers (CHWs) playing major roles [49], and with power devolved to communities [50]. In practice, however, there is slow and uneven implementation of WBPHCOTs, low coverage, insufficient staff, and low awareness of expanded CHW roles [51–53]. Mandated participation in health is also limited. Beyond representation on clinic committees, community participation is confined to basic clinic support [54–56]. Frontline providers are similarly reported to experience top-down bureaucracy and ‘compliance cultures’ undermining their ability to respond to local needs [57, 58]. Implementation challenges notwithstanding, South Africa delivered a community-based response to COVID-19. In 2020, 28,000 CHWs were deployed across the country for community screening and door-to-door testing and contact-tracing [59].

### Objectives

There is an urgent need for practical understandings of how to build and sustain community power in PHC for the attainment of health equity. The objectives of the study were to (a) conduct a theory-informed analysis of community power-building in PHC in a setting of structural deprivation, and (b) develop practical guidance to develop the process as a sustainable PHC component.

## Methods

### Study design and setting

**Fig. 1:**
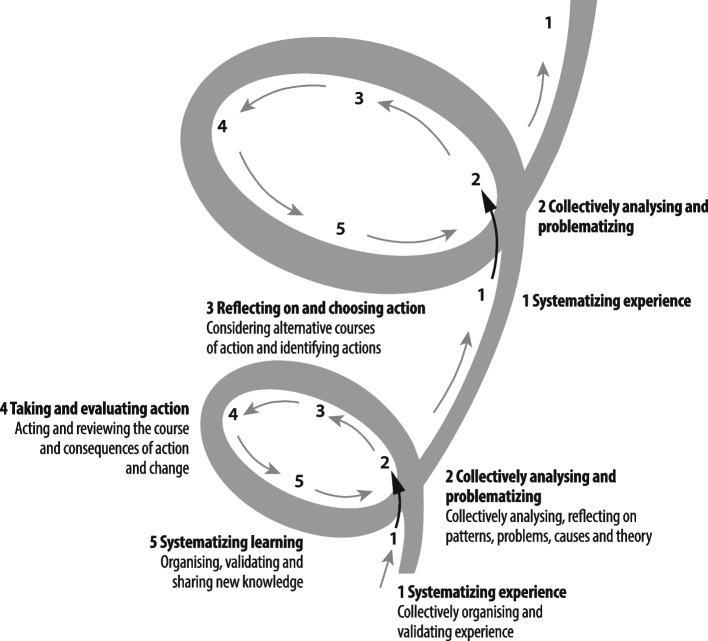
The cyclical process of PAR (source [30])

**Fig. 2:**
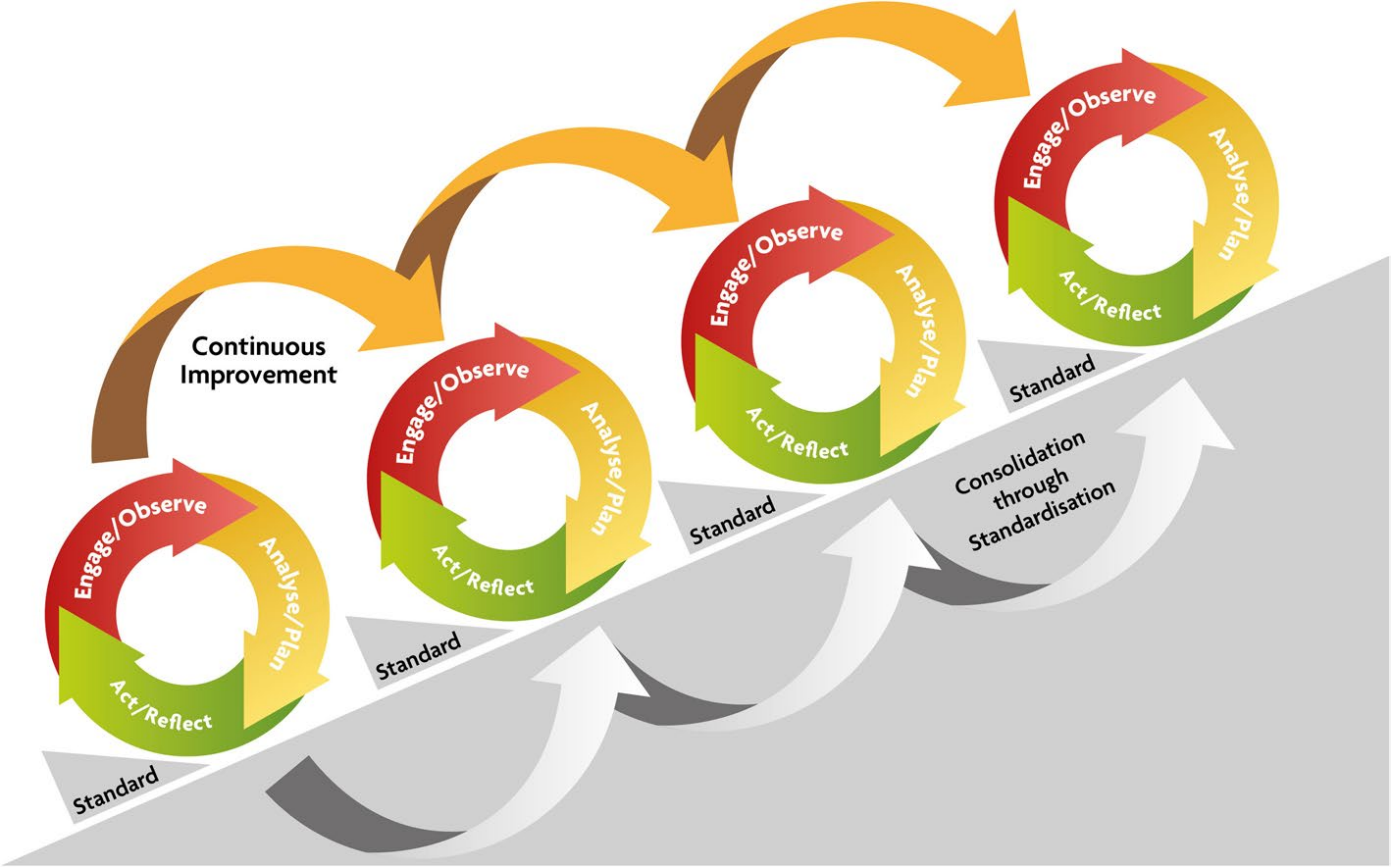
VAPAR action-learning cycles

**Fig. 3:**
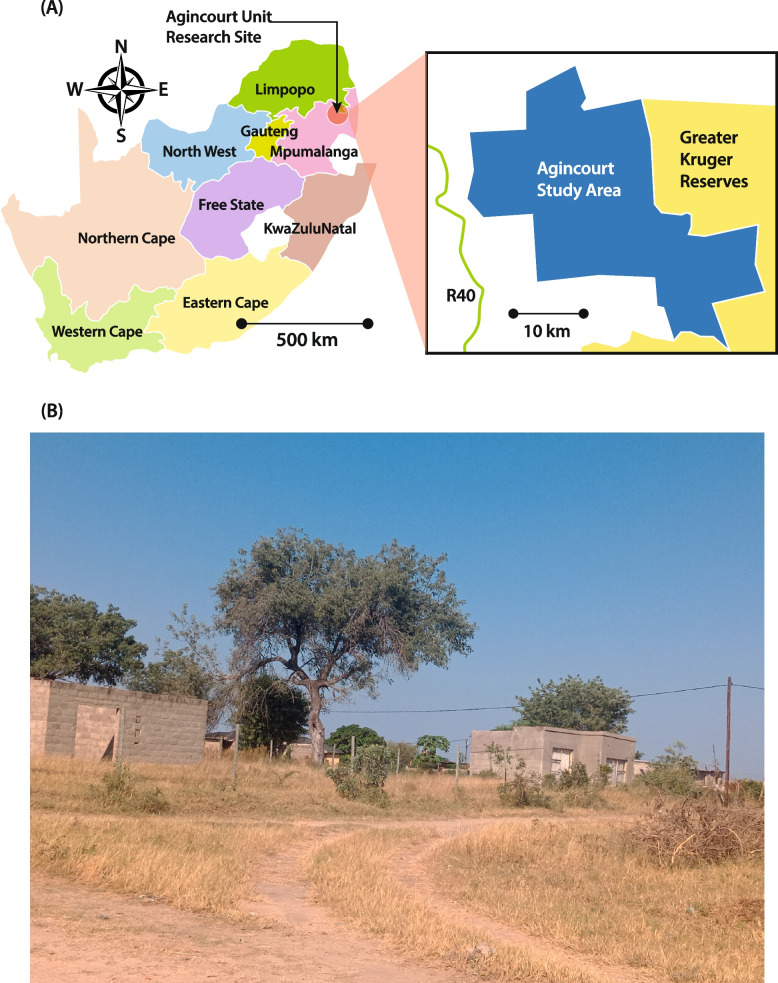
**a** Map of Agincourt Health and Socio-Demographic Surveillance System (HDSS) (**b**) village within Agincourt HDSS

The PAR process was part of the Verbal Autopsy with Participatory Action Research (VAPAR) programme (www.vapar.org) [60]. As described above, PAR emphasises local expertise, democratisation of knowledge production and empowerment through collective action-learning. PAR reframes the roles of those participating as active researchers and change agents and is concerned with collective action as a means to new knowledge [30] (Fig. 1). The programme was organised around a PAR framework with series of reiterative cycles connecting service users and providers to generate and act on evidence of practical, local relevance. Each cycle had three components: ‘engage/observe’, ‘analyse/plan’ and ‘act/reflect’ (Fig. 2). The design was rooted in health policy and systems research (HPSR), focussing on how societies organise to protect and promote health, and health systems as complex, adaptive, human, and relational [61, 62]. The research was based at the MRC Wits/Agincourt Health and Socio-Demographic Surveillance System (HDSS). Established in 1992, Agincourt is one of southern Africa’s largest and oldest population cohorts, covering a population of 120,000 over 450km2 and 31 villages [63] (Fig. 3). In the area, rural homesteads experience multigenerational deprivation. There is little formal sanitation, unaffordable electricity, high unemployment, and a limited economic base resulting in labour migration and reliance on social grants [64].

### Participant recruitment and data collection

#### Cycle 1 (2017–19)

In 2017, we approached participants involved in pilot work 2015–16 (*n* = 24) across three rural villages. Villages had been selected to vary by socio-economic status and access to services. Within each village, participants were selected to represent community actors, service users and providers. Nine participants were unable to continue, and new individuals were recruited. Introductory/codesign workshops were held in which priority health issues were identified, and in which participants directed expansion of the participant base to bring in the perspectives of those most directly affected by the issues identified, and whose voices were most excluded. Additional participants were recruited on this basis (*n* = 24). A series of ‘engage/observe’ workshops of 2–3 h each then progressed in each village and subsequently bringing groups together (*n* = 16 workshops, 48 participants total). Workshops were sequenced to: (a) build shared understandings of problems, (b) appraise contexts and impacts, (c) develop action agendas, and (d) reflect on and adapt the process. We also used photovoice to collect and analyse visual evidence (Additional file 1).

In weekly workshops, we (re)negotiated PAR principles of shared interests and concerns, subjective experience, collective validation, and no delegation (Table 1). Participants directed practical aspects like times and locations of meetings. We also collated and analysed VA data to quantify mortality associated with community-nominated priorities and reworked VA to capture social circumstances of deaths. In 2018, representatives from the authorities came together with community stakeholders in the ‘analyse/plan’ and ‘act/reflect’ components (*n* = 15–18 participants). Through six further workshops over a six-month period, we collectively appraised and analysed VA and PAR data and evidence, and developed, and implemented local action plans (Additional file 2). Following a four-month implementation and monitoring period, all stakeholders collectively reflected on the process, and made adaptations to improve local relevance and practical utility.

**Table 1:** PAR Principles (source: [53])

Principle	Description
No delegation	Participants are those directly affected and are the primary researchers taking lead roles forming teams to identify problems, define, analyse, and develop solutions
Homogeneous group	The focus issue should be deliberated over, and consensus on the nature of the problem and actions to address the issues identified among a social group with shared conditions
Subjective perspective	People’s individual experience are central to the process and are the foundation upon which collective knowledge is developed, respecting each other’s opinion, as opposed to imposing ideas/opinions on others
Collective validation	Recording observations that all participants identify as important but does not negate differences in perception and experience, but the group must reach consensus on collective findings. Corroboration of information and experiences

#### Cycle 2 (2019–20)

In 2019, we re-engaged community stakeholders to progress the second cycle. Deaths, migrations, and other commitments precluded involvement for some, and new participants were recruited (*n* = 10) however when ‘word spread’ of the second cycle, additional original participants re-joined (*n* = 53 participants total). We held a further series of village-based workshops building learning, insights, capabilities, and connections with formal PHC governance within and between the three villages (*n* = 16 workshops in total, following the sequence in Cycle 1). We progressively expanded community ownership and control with participants, who had become more familiar with the processes and tools, directing and facilitating meetings. From early 2020, in-person workshops became impossible due to lockdown restrictions. In response, we interrupted the cycle and collectively redesigned it. Using telephonic and other remote engagements we engaged with senior government officials (*n* = 11), local health systems actors (*n* = 38), rural dwellers, community-based providers (*n* = 90), and local interagency COVID response fora (*n* = 2) to gain insights into how the research could support the district pandemic response. The consultation revealed a collective realisation of CHWs as critical to the COVID response, serving as a link between communities and services, but lacking in support.

#### Cycle 3 (2021–22)

On this basis, the subsequent cycle was reconfigured to include a training programme in ‘rapid PAR’ supporting CHWs to develop community mobilisation competencies. The training focussed on convening community stakeholder groups, raising and/or responding to priority health concerns, understanding the nature of concerns from different perspectives, and initiating dialogue on and facilitating and monitoring action in communities, the health system, and public services. A training manual was prepared to support the process (Additional file 3). In late 2020, the approach was discussed with operational managers (OMs) from three clinics serving the villages in which prior cycles had progressed to ensure relevance and practical utility. Invitations were then extended to CHWs from the three clinics to join the training programme. OMs and researchers selected three CHWs from each clinic based on skills, motivation, and interest (*n* = 9 total).

To preserve community control, three community stakeholders from the previous cycle agreed to join the cycle as ‘community mentors’ to provide support and insight into participatory principles. To further locate the process in the contexts and needs of WBPHCOTs, three initial workshops brought together PHC staff, CHWs, community mentors, OMs, and clinic committee members from the three clinics. In the workshops, researchers introduced the participatory nature of the training, the emphasis on sharing power and control and building shared ownership over local action. Through facilitated discussion, participants nominated priority health issues, and ranked these using voting. The workshops provided orientation to and training in PAR to collectively problematise the nominated health issue, to collect visual data, and on facilitating deliberative group discussions.

CHWs purposively recruited a further nine community participants from each village (*n* = 27 total) according to two criteria: (1) individuals directly affected by, and with key insights on, the issues under investigation, and (2) individuals whose voices might be otherwise excluded from planning and action on the issues identified. There were no refusals in the recruitment of CHWs, community mentors and community stakeholders.

Weekly workshops then progressed in each village-based clinic area. Over 10 weeks and 15 workshops, the training progressed through a similar sequence to previous cycles. CHW and community stakeholders nominated priority health concerns, identified relevant stakeholders, connected with actors at higher levels of the system and developed, implemented, and evaluated local action plans (Additional file 4). Finally, we engaged with planners, managers, and frontline providers to reflect on and refine the process.

The elements outlined above—pilot work [65], Cycle 1 community-based process [66–68], multisectoral engagement [69], VA [70, 71], reflective/adaptive element [72], Cycle 2 redesign [73, 74] and Cycle 3 CHW training [75]—are reported elsewhere.

### Data analysis

We used the Emancipatory Power Framework (EPF) to understand community power building [76]. The framework consists of three power perspectives through which capabilities for collective control and change can be understood. ‘*Power within’* refers to collective capabilities internal to a community, including recognition of shared interests and values; ‘*power with’* encompasses power evolving when communities work with other agencies/communities; and ‘*power to’* refers to collective capabilities associated with implementation of community action, including the establishment of structures and opportunities for collective action. We also used the Limiting Power Framework (LPF), sensitive to spatial dimensions of negative power within and beyond the ‘local’. These two frameworks encourage attention to both the ‘inward’ and ‘outward’ gaze – i.e., on community capacities, and on social and political transformation for greater equity [76].

Data sources included recordings of workshops, researcher notes, visual and VA data, and formal and informal stakeholder feedback. An immersion/familiarization process and review of data was followed by thematic content analysis [77]. Analysis related emergent themes to categories derived from the EPF/LPF as well as recording unanticipated themes. This progressed until a reasonable point of thematic saturation was attained. Analysis was mainly electronic: using NVivo software, accompanied by written and verbal team exchanges.

### Ethical considerations

Ethical conduct was considered in terms of control over the process, fair benefit, inclusion, and power sharing. Potential power imbalances were acknowledged, considered, and re-visited throughout, as was respect for traditions, languages, and values of participants. All participants provided written informed consent. Potential consequences were explained prior to involvement, participants could withdraw from the process at any time, were provided with written information on the research in the local language and given minimum 72 h to absorb and ask questions before agreeing to be involved. Participants were provided with refreshments, transport costs, and reimbursed for time spent participating. All identifiable data were anonymised. Explicit permission was secured from participants for the reproduction of images taken during the research. Data were stored on secure servers hosted by the Agincourt HDSS and the University of Aberdeen, managed, and analysed using NVivo version 12. Institutional review boards at the University of the Witwatersrand (M1704155; M171050) and University of Aberdeen (CERB/2017/4/1457; CERB/2017/9/1518) reviewed and approved study protocols and the provincial health authority gave permission for the research (MP_201712_003).

## Results

The analysis is arranged by the EPF and LPF to illustrate power dynamics, how community power built and was sustained within and between cycles, and overall. The results are illustrated by verbatim quotes, visual data, and reflections from community, and health systems stakeholders and researchers.

### Cycle 1: establishing collective capabilities and spaces with mutuality, and collaboration

In Cycle 1, community stakeholders identified alcohol and other drug (AOD) abuse and lack of safe water as priority health concerns. Youth and women of reproductive age were nominated as people affected by and whose voices were excluded from attention to the issues respectively. Including these perspectives was fundamental, however some youth participants directly affected by alcohol and drugs were disruptive, aggressive, uncooperative, and despondent about the possibility of change. Sensitive and assertive facilitation was required to ensure the inclusion of those most directly. We were sensitive to pessimistic viewpoints, and reiterated principles of respectful conduct and representation, and expectations for change were managed carefully, with honesty and consistency.

**Fig. 4:**
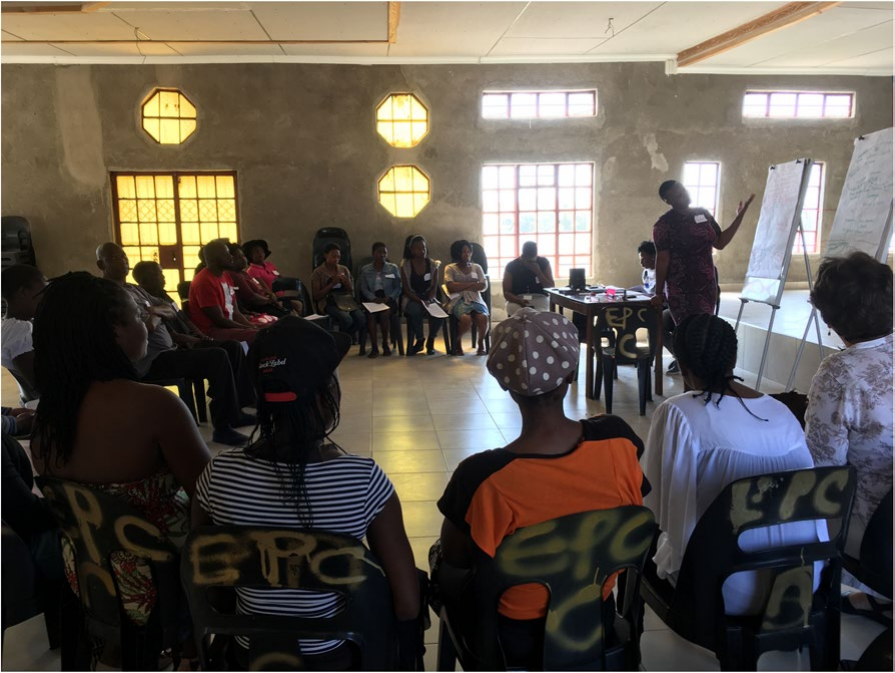
Community stakeholders leading workshop discussions and analyses. Permissions have been secured from participants for the reproduction of all images taken during the research

Engagement gradually improved as core principles were transmitted, discussed, revisited, owned, and taken up. Ownership was supported as participants assumed control of the process: identifying priority health concerns, directing expansion of the participant base, and controlling practical aspects such as, dates, times, and venues of workshops. Collective capabilities, ‘*power within’*, developed as participants’ familiarity built with public speaking, analysis (including with causal maps and in selecting, appraising, and captioning visual data), consensus-building, and in co-facilitation and recording of meetings (Fig. 4). Regular revisiting of PAR principles moreover supported shared vision and purpose. More coherent, respectful exchanges emerged as a result, with quieter participants speaking up and more dominant participants giving space for others to talk:

*“…we should talk about one actor at a time because others talk about the pastor while some talk about police and it is confusing” (Woman of reproductive age, Cycle 1).*

*“…I want to say that in the past we were laughing to each other when someone talks but now there is a change. We are united, we listen to each other” (Community stakeholder, Cycle 1)*

Initially, each village represented themselves but over the course of the workshops, groups realised they had common issues and worked together. As a weekly rhythm of workshops was established, groups collectively deliberated over causes and impacts of AOD abuse and lack of water, mapped key stakeholders and agencies, and developed and appraised local action agendas. ‘*Power within’* developed further; community stakeholders reported becoming more informed about local issues, processes, and structures, and collectively learned ways to address shared concerns.

*“…we gain knowledge, we learned about caring for ourselves and to work together with other people” (Community stakeholder, Cycle 1)*

Unemployment, poverty, and proliferating taverns were identified as key drivers of AOD abuse, which was conveyed as destructive of communities, and disproportionately affecting children and young people [66, 68]. On water, repeated and prolonged periods without piped water documented, as were unreliable and unavailable infrastructure, inadequate service delivery, unregulated sources, empty reservoirs, and poor supply exacerbated by droughts [67]. We supplemented community intelligence with statistical data on the extent of the burden and its social and circumstantial drivers [70, 71].

Credible, actionable information, and collective capabilities were the foundations upon which we engaged with the authorities. Reflecting the community-nominated priorities, we engaged widely with different levels and sections in Departments of: Health; Water and Sanitation; Basic Education; Cooperative Governance and Traditional Affairs; Social Development; Home Affairs; Culture, Sports, and Recreation; the Local Drugs Action Campaign; Water Catchment Management Agency; with non-government stakeholders such as the South African National Council on Alcoholism and Drug Dependence (SANCA) and the Africa Foundation.

**Fig. 5:**
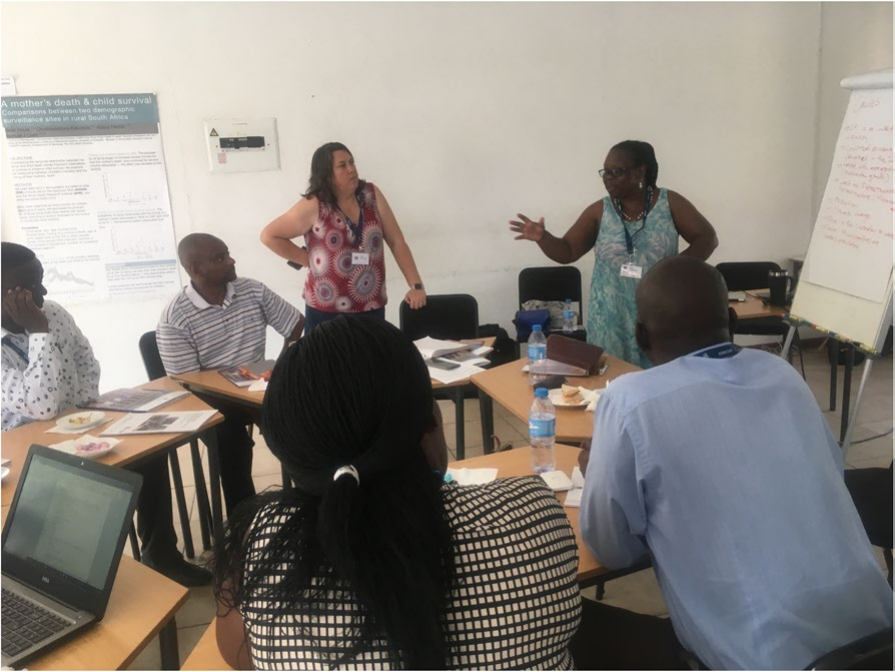
Multisectoral action planning workshop. Permissions have been secured from participants for the reproduction of all images taken during the research

There were new and varied power dynamics in these workshops and some discussions were dominated by powerful local officials. Again, we revisited PAR principles regularly, and with sensitive/assertive facilitation, constructive and respectful dialogue was supported. Through this process, ‘*power with’* emerged; health officials came to see and welcome a view of community stakeholders as active change agents, rather than passive beneficiaries, and criticism of the authorities from communities gave way to a collective awareness that working in isolation would not support solutions. Community power deepened as tangible commitments for local action were developed with representatives of the authorities (Fig. 5):

*“There have been a lot of service delivery protests in communities, but they did not accomplish much – everyone realised that it is time to shift our ways of thinking and initiate dialogue, unite and collaborate and create sustainable partnerships to solve community problems” (Community stakeholder, Cycle 1)*

*‘Power with’* deepened as stakeholders developed and collectively implemented local action plans. There was mixed success with the action plans (some actions were achieved, some partially, and some did not progress). As the researchers monitored action through follow-up visits, entrenched systems challenges became evident [78]. In response, we framed the monitoring process as a learning opportunity: prioritising psychologically safe spaces for reflection in a no-blame, appreciative approach. This supported acceptability. In the final reflection element of Cycle 1, the authorities embraced the process as a platform to safely engage with communities without having to defend themselves, and for multisectoral dialogue. Health officials proposed adaptions to deepen links between researchers, communities, and health system: integrating the process as a standing item in district health management team (DHMT) meetings and with local health officials joining the PAR community workshops [72]. This deepened opportunities and spaces for collective action and conferred a new legitimacy to the process, enabling *‘power to’*.

### Cycle 2: Expanding spaces for local decision-making

**Fig. 6:**
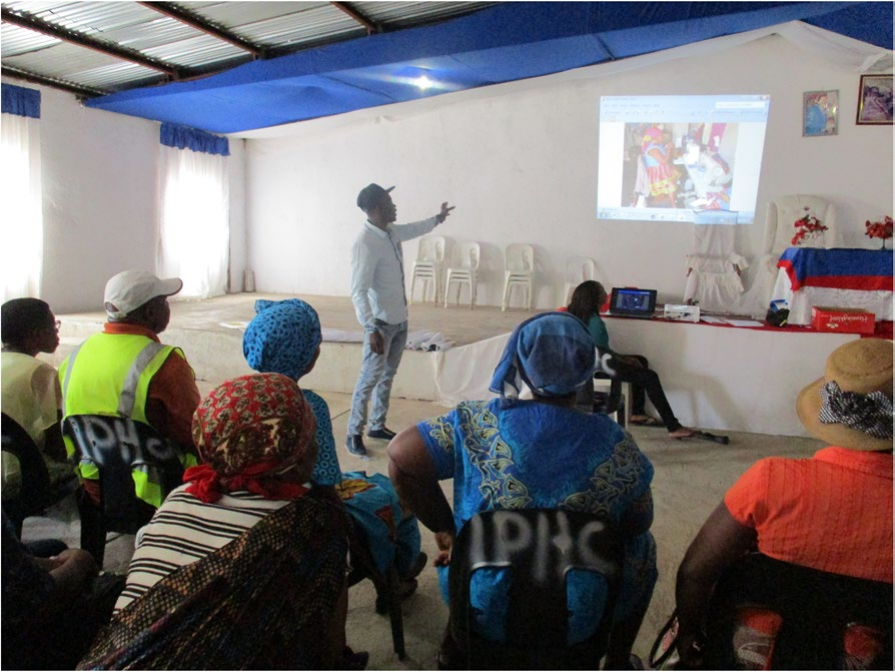
Expanding who participated and sharing control built collective capabilities. Permissions have been secured from participants for the reproduction of all images taken during the research

Grounded in and accepted by the district health system, a pronounced ‘localisation’ emerged during the second cycle. Community stakeholders engaged more deeply as co-researchers. Community ownership extended further as participants identified and recommended new stakeholders to join. As well as those directly affected, this included leaders from the traditional authorities and the Community Development Forum (CDF), which supported more nuanced understandings of local power structures and dynamics. In the development of action agendas, fewer actions were developed, and they were more locally focussed and achievable. Community stakeholders committed to act locally and to build partnership with the local governance in villages. Community stakeholders, moreover, worked with the PAR tools quickly as they understood them, taking ownership of discussions, leading, and facilitating the deliberations (Fig. 6). This deepened ‘*power within’* and extended ‘*power with’:* identifying key actors affirmed stakeholders using their voice and courage to reach out and connect. Creating partnerships and capacity and building new connections in local services and across sectors further enabled identification of, and engagement with, those with the power to act.

Regular interaction with a range of stakeholders revealed few spaces for departments to engage, and the process was again seen as valuable among representatives of the authorities. Participants co-designed time-bound action plans enabling accountability from different departments with more focus on local stakeholders. Community stakeholders tailored demands while fostering strategic relationships. While some dissatisfaction with the authorities remained, constructive mind-sets were also evident with deeper understanding and appreciation of structures, opportunities, and responsibilities for collective action:

*“…we really understand…. that they won’t fix the water problem overnight… what matters is that we have already taken the step of approaching people who we know are dealing with water. This is us taking responsibilities for our problems.” (Youth participant, Cycle 2)*

*“We can use the methods to solve community issues, for example when there is no water or electricity people may decide to strike where they usually destroy infrastructure but now that there are people like us who have been exposed to these methods we can opt and encourage everyone to use this approach since it is peaceful and attracts attention.” (Community stakeholder, Cycle 2)*

As COVID took hold in early 2020, the second cycle was interrupted and redesigned to be of practical support in rural communities and the district. Through remote and in-person consultations during lockdown, rural communities and service providers expressed concerns over lack of understanding of preventative measures to reduce transmission, and about access to non-COVID care. There was a shared realisation that CHWs were the first line response to connect people and services, especially vulnerable people, but lacked support. The collective re-design revealed an urgent need to formalise dialogue spaces for collective action. Both community stakeholders and service providers realised the gap/disconnection between them, and collectively agreed to fill the gap with the learning platform (Table 2) [74, 73]. This extended ‘*power to*’ in terms of establishing new structures, processes and opportunities cooperative learning and action.

**Table 2:** Priorities and concerns of communities, service providers and CHWs in the context of the pandemic

Priorities and concerns / Key constituencies	Community	CHWs	Service providers
Fear over pandemic in communities	X		
Lack of water remains pervasive undermines handwashing	X		
Sanitisers expensive	X		
Resolution of noise, accidents, and violence with alcohol ban	X		
Myths and misinformation in communities and on social media	X		
Financial impacts of lockdown, food insecurity/child malnutrition	X		
Lack of understanding in communities on preventative measures	X		X
Feasibility of preventative measures in communities	X		X
Various application of preventative measures in communities	X		X
CHWs well-placed to support preventative measures	X		X
Access to non-COVID care challenging	X		X
Urgent need for research on community voice	X		X
Need for clarity with CHW programme and roles	X	X	X
Need for dialogue and partnership services and communities	X	X	X
Need for support for CHWs to engage rural communities			X
Reliable local-level data routinely available			X

### Cycle 3: Connecting and sustaining structures and opportunities for action

The third cycle was reconfigured to include a training programme to support CHWs to develop community mobilisation competencies by connecting, raising, and responding to local health concerns, using rapid PAR tools and techniques, and facilitating action in communities, the health systems, and public services. While CHWs had relationships within communities, processes to convene to discuss issues faced by the community were not optimised and often disrupted [56]. Training CHWs in PAR methods and principles for bottom-up learning and action, and embedding platform in community health system, was seen as worthwhile in this space.

In the third cycle, CHWs led a collective decision to focus on attitudes, interactions, and behaviours between communities and services, specifically focussing on people lost to follow up with HIV/TB treatment as a critical and potentially overlooked area as services shifted to COVID. Issues were problematised and local action plans collectively developed, implemented, and monitored. Action plans were again focussed and local: to improve access to information, support, treatment, and care including through support groups addressing stigma and related priorities such as food security and access to social workers.

*‘Power within’* was again built. Community mentors supported CHWs during the training; building capabilities, improving public speaking and application of PAR tools. Community mentors also supported CHWs managing dominant participants, listening to, and respecting everyone regardless of status or power. CHWs and community mentors also took it upon themselves to arrange venues for workshops, remind community stakeholders about workshop logistics, and in facilitating and recording activities during action plan implementation. Collective rather than researcher-led monitoring further strengthened CHW and community ownership and control.

**Fig. 7:**
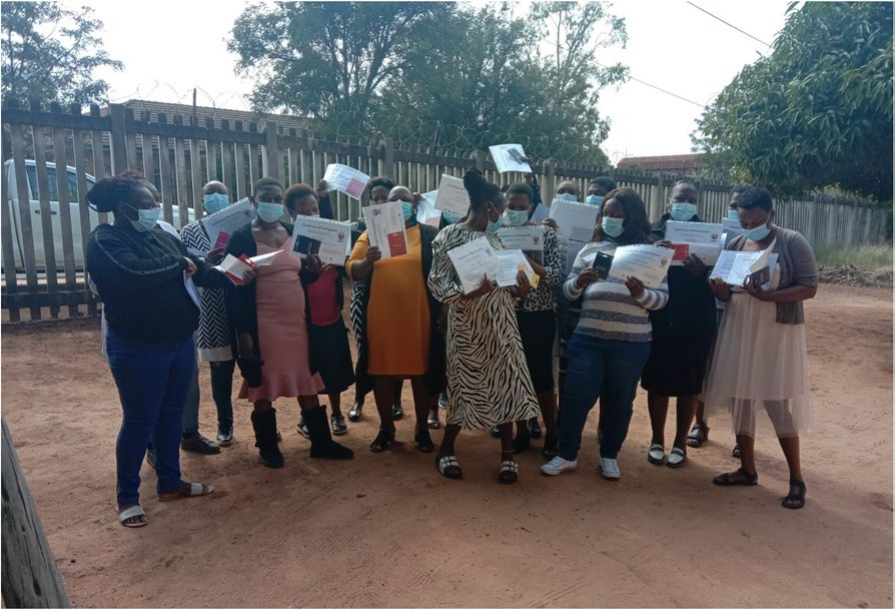
Completion of the CHW PAR community mobilisation training course. Permissions have been secured from participants for the reproduction of all images taken during the research

*‘Power with’* was seen in terms of commitments to the process throughout community and clinic levels of the system: clinic Operational Managers (OMs) and outreach nurses (OTLs) were highly supportive throughout, regularly attending meetings to demonstrate support by ‘voting with their feet’ [79]. This also fostered ‘*power within’:* CHWs reported that the process made them feel recognised by clinic staff, and programme managers and other officials, and that they felt respected and valued. CHWs also reported building new CHW peer-alliances, indicated that their presentation and reporting skills improved significantly, and that they were more determined to be involved in the communities [79]. Through the PAR training, quality relationships between CHWs were also seen as both a positive experience and strategic benefit (Fig. 7).

*“The VAPAR training was good…I learned a lot about respect, communication and how to use all the tools that we learnt during the training. Most importantly, I learnt the power of working together as CHWs, communities and traditional authorities. If the communities can master this approach of working together, we can solve a lot of issues that our communities come across every day.” (CHW participant, Cycle 3)*

*“The training taught me ways of identifying challenges and addressing them, I understand challenges better than I used to. I'm confident that now I know even how to identify people who can assist us in dealing with various issues.” (CHW participant, Cycle 3)*

*“I can use the skills I learnt during the training to work with community members and other CHWs to identify the challenges we have and work together to solve them.” (CHW participant, Cycle 3)*

*‘Power with’* was further built, following completion of the CHW training, as the process progressed to engaging with higher levels of the system to analyse, interpret, act on, and learn from the data and evidence generated. Again, departments realised that each have policies that address common problems, but that these are disconnected. The learning platform provided a process for stakeholders to convene, discuss and collectively find new ways to address issues faced by communities e.g., via clinic committees supporting CHWs with guidance on where and how action can be implemented. Clinic OMs and higher-level managers became integral to the process, supporting CHWs and participating.

Engaging with actors in health system was challenging, with many disruptions owing directly and indirectly to the pandemic. We sought to be flexible and sensitive, and worked to avoid imposing administrative and time burdens in an already over-burdened system. We explicitly discussed practical relevance and benefit at several points, and with reference to systems constraints and unanticipated demands from COVID-19. This further supported trust relationships. Crucially, as the process engaged sensitively and authentically with the authorities at higher levels, there was explicit recognition of CHW roles, value, and contribution.

*“I never knew how much these people [CHWs] know…we never knew we could learn from these people” (Health system manager, Cycle 3)*

*“For the first time, CHWs and managers sit at one table and engage” (Researcher, Cycle 3)*

*“The [VAPAR CHW training] manual, from the department’s perspective, particularly at the sub-district level, inspires a great sense of pride about the realisation of the possibility of building capacity for this cadre of emerging health care workers in South Africa. The manual will go a long in providing a practical and a formal tool to guide CHWs through their day-to-day work with communities” (Health system manager, Cycle 3)*

At the end of the third cycle there was again a collective reflection. There was agreement to work to re-establish a CHW support structure that had previously existed with clinic outreach teams, and to embed the PAR process into clinic processes. This was welcomed by OTLs who did not see the process as imposed but as one that was owned by and relevant to WBPHCOTs. The shift was formalised in a request to roll out the community mobilisation training through the sub-district with the Department of Health (DoH). The third cycle thus drove ‘*power within’, ‘power with’* and ‘*power to’*; developing a collective voice driven by CHWs, connecting with other agencies and communities, and by further embedding the platform with a focus on CHWs as key public health agents in the district health system, respectively. Analysis with the EPF revealed that power-building dynamics were non-linear; different components of the EPF progressed in different ways in each cycle, and overall (Table 3).

**Table 3:**
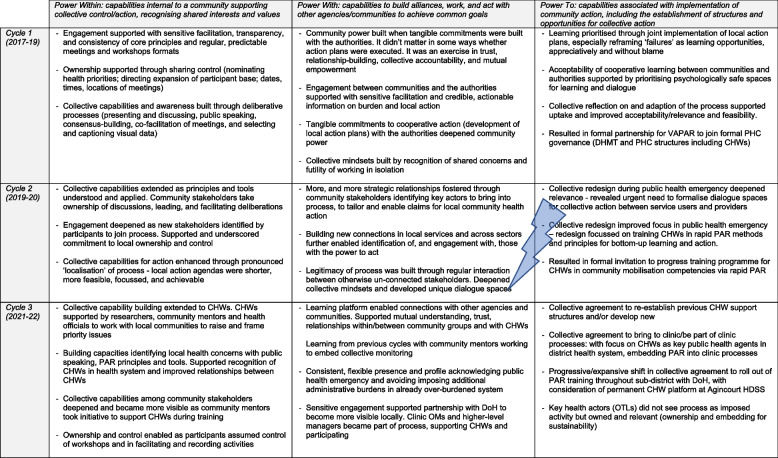
Emancipatory power building: power dynamics observed within and between action learning cycles

Key:  Emergence of COVID-19 in early 2020

### Limitations on power building

We also used the LPF to document forms of power limiting community control, measures to address these and areas that need attention. *Compulsory power* is defined as direct and visible exercised through, e.g., legislation. Several formal and restrictive dynamics were seen, for example, community voice expressed in illegal service delivery protests, a criminalising approach to AOD, and in the strict lockdown. Compulsory power can be seen to limit community voice, and contextualises the value expressed by stakeholders for safe dialogue spaces between service providers and users, for reframing AOD abuse as a public health concern, and for driving attention to and action on community health priorities, when these were limited owing to COVID.

*Institutional power* is defined as less visible, exercised through organizational rules, procedures, and norms. Here, we observed top-down governance, limited learning spaces, and little recognition of the significant capability, resilience, and ingenuity at operational levels of the system [78]. There was little recognition, initially, of community members as active change agents, poor functionality of participatory governance structures and processes such as clinic committees, and some discussions were dominated by local actors. We also observed parallel systems and tensions between groups such as the CDF, which had varied roles, and with traditional authorities and former tribal areas dominating democratic spaces, which on some occasions limited community voice. We strategically positioned the process to increase visibility and legitimacy—building alliances with and influencing formal structures and actors e.g., DHMT. Nevertheless, some actors at higher levels of the system had limited power to act owing to institutional power limiting dynamics.

*Structural power* is defined as invisible, systematic biases embedded in social institutions, generating/sustaining social hierarchies of class, gender, ethnicity and resources, opportunities, and social status. The second and third cycles revealed the centrality of CHWs to the DHS, and especially in the face of emerging threats such as COVID-19. Building collective voice and agency with CHWs revealed that, despite this centrality, in practice CHWs experience multiple challenges: lack of financial, logistic and health system support and training, lack of role clarity, insecure employment, low and no pay, poor safety, and low status [75]. We observed engrained social inequalities and hierarchies along various lines—ethnicity, gender, class, occupation (especially with CHWs)—and that these acted in combination with e.g., institutional power dynamics.

*Productive power* is also invisible, operating through social discourses and practices to legitimate some forms of knowledge, while marginalizing others. The wider context of the learning platform, in former homelands characterised by intergenerational structural disadvantage, economic inequalities and enduring stigmatisation of HIV/AIDS was important. During workshops, some stakeholders were dominant and disruptive, leading to others feeling intimidated to raise their opinions. In some instances, we observed local politicians using the platform to promote political parties. We dealt with this with sensitive, but assertive, facilitation reinforcing principles of democratic participation, voice, representation, and respect. The regular negotiating of these principles supported more equal participation. Finally, in some clinical and academic spaces, we observed views of enquiry paradigms concerning knowledge for action, plurality of knowledge, and expertise from the margins as low quality and/or non-science. We dealt with this with a consistent presence, open and reproducible methods and engaging widely in critical debate and activity across disciplines and sectors (Table 4).

**Table 4:** Limiting power framework: power limiting factors and forces observed during Cycles 1–3

	*Compulsory power ‘direct and visible’:* *Direct and visible exercised by/through, e.g., police, local and national legislation*	*Institutional power ‘less visible’:* *Exercised through organizational rules, procedures, and norms, e.g., controlling information put into the public sphere, who is involved in decision-making*	*Structural power ‘invisible’:* *Invisible, systematic biases embedded in social institutions, generating/sustaining social hierarchies of class, gender, ethnicity and resources, opportunities, social status*	*Productive power ‘operates through practices’:* *Invisible, operates through diffuse social discourses and practices to legitimate some forms of knowledge, while marginalizing others. Shapes meanings of different social identities*
Power limiting dynamics	- Community voice typically expressed through violent service delivery protests - Alcohol and drug abuse criminalised and stigmatised, less of public health response. Leads shift away from public health approach with collective action - Legislation shifting the system to COVID responses limited attention to other critical public health priorities	- Top-down governance. Pronounced ‘compliance culture’ in district health system. Limited spaces for learning, little recognition of resilience, ingenuity that exist at operational levels of the system – marginalises experiential knowledge - Poorly functional participatory governance. Limited, initial, recognition of community members as active change agents - Discussions open to capture by dominant or powerful actors - Parallel community governance systems and tensions. CDF varied roles within – limits potential. Traditional authorities and former tribal areas dominating democratic spaces, limits community voice	- Local politics seen in some discussions dominated by local politicians - Little recognition of expertise from the margins, little recognition of alternative forms of data and evidence (e.g., visual data) (academic institutions) - CHWs experience multiple challenges: lack of financial, logistic and health system support and training, lack of role clarity, insecure employment, low and no pay, poor safety, and low status - Deeply embedded social inequalities (ethnicity, gender, class, occupation), combines with institutional biases to marginalise some voices and privilege others	- Rural village contexts – former homelands direct/visible implications around inadequate infrastructure/ racial segregation endures. Economic inequalities—generational impact - HIV stigma endures. Systems structuring reinforces stigma in how services organised HIV/AIDs and TB remain deeply stigmatised - Legitimizing certain forms of knowledge and knowing over others – creates stigmatising identities for people experiencing hardship
Forms of resistance/Areas that need attention	- ‘Safe spaces’ for dialogue between communities and authorities - Reframing of AOD abuse as public health priority - Process grounded in community needs and realities highlighted attention to HIV/TB treatment continuity, as services shifted to COVDI-19 responses	- Process claimed and protected spaces for new forms of ‘everyday leadership’ rooted in community voice, service response and data and evidence provision - Process strategically positioned to increase visibility and legitimacy—building alliances with and influencing formal structures and actors e.g., DHMT (district level), training resources (sub-district) OTLs and OMs (Clinic and community level) - More attention needed to enable and recognise significant resilience, capability, and ingenuity at operational levels of the system	- Consistent presence, navigating many different worlds to challenge established narratives around voice and power - Cooperative, appreciate approach to sensitive conversations, building of alliances, relationships, and trust - Working to strengthen platform to build collective power in different worlds: community, health system, academic spaces	- Sensitive and assertive facilitation, working to avoid imposing additional burdens in an already overburdened system - Dominant and stigmatising narratives challenged through consistent, predictable, transparent presence. ‘Neutrality’ conferred by virtue of connection to research/data centre - Use of different forms of media e.g., radio, podcasts to create positive narratives about community action and community health (narrative resilience)

## Discussion

In this section, we discuss the development of community capabilities (with reference to Popay’s ‘inward gaze’), and wider systems and structural influences on these (in terms of Popay’s ‘outward gaze’). Finally, we reflect on and summarise key insights and transferrable learning.

### Establishing and expanding learning spaces (‘inward gaze’)

International evidence indicates that social power in health is strengthened when grounded in community settings [80]. ‘*Power within’* was built by grounding the process in rural village settings where we progressively expanded community control through regular engagement prioritising respect and connectedness. Collective capabilities developed as participants nominated local health concerns and directed who participated, where and when, eventually facilitating the discussions. Nomination of water and AOD abuse moreover connected the process to sectors beyond health and revealed entrenched problems.

When grounded in community settings, participatory processes can also account for social and cultural challenges and beliefs, and address stigma and social isolation [81]. The development of collective mind-sets was evidenced in realisations of shared interdependencies and mutual vulnerabilities. While efforts were made to connect with ‘hard to reach’ groups and those most directly affected, however, assumptions about who disadvantaged communities are, especially those of outsiders, can be problematic [82]. To deal with this, continual attention to and reworking of representation, in locally appropriate and relevant terms directed by community actors, is a priority to avoid reinforcing the interests of the already powerful.

Community power was further built by adapting and expanding the process with service and systems actors. This allowed for a local process to connect with institutional structures and processes. ‘*Power with*’ manifested when community stakeholders collectively developed and implemented local action plans with representatives from the authorities. ‘*Power to*’ built as partnerships formalised and the process was taken up by the DHS developing new opportunities, spaces and processes for collective action and learning. This enabled new possibilities to engage with wider systems and structures to address local health concerns, encourage mutual accountability and raise awareness about services within and beyond health, including with NGOs.

### Connecting and sustaining learning spaces (’outward gaze’)

Engaging those with the power to act was crucial. Wider debates acknowledge that exclusively ‘local’ processes may be limited, based on assumptions that problems are only local [83, 84]. We engaged widely, within and beyond the health sector, and managed forms of power that were more and less visible in psychologically safe spaces. This deepened insights into organisational contexts, and how to navigate them. Bringing together community and multi-sectoral stakeholders was challenging, however. Although multisectoral working in health is widely advocated, practicalities are not well-understood [85–89]. Nevertheless, when ‘safe to fail’, community stakeholders realised shared capabilities to use their voice, while service providers came to see the platform as a safe way to engage, and collective mindsets were built. These relationalities should not be underestimated; there are frequent, violent service delivery protests in the area especially over water [90, 91]. The process shifted otherwise disconnected actors towards connection and constructive dialogue.

In the third cycle, the focus shifted to CHWs roles, functions, relationships with communities and HIV/TB treatment in the context of the early part of the COVID-19 pandemic. While this was a departure from sole community control, it extended mutuality between community-based service users and providers in the initial stages of a new public health emergency. COVID-19 had severe impacts in local neighbourhoods, adding to an already complex, dynamic, and deeply unequal disease burden, devastating lives and livelihoods [46, 59]. While a highly centralised response was initially effective in controlling spread, CHWs engaged in the process expressed serious concerns about diagnosis and treatment of other conditions, particularly HIV/AIDS and TB, and over food security. These concerns have since been echoed nationally and internationally [59, 92, 93]. The process enabled CHWs to raise concerns during interactions with sub-district health managers and other stakeholders within the PAR gatherings, during action planning and monitoring. This grounded health priorities in local realities and supported recognition and attention to them among those with some degree of power to act.

As we worked with CHWs, multiple institutional and structural biases were revealed. Related to ethnicity, class, gender, and occupation, together these severely undermined CHWs’ functionality [79]. CHWs are a critical workforce in decentralised health systems, uniquely positioned to provide accessible, culturally appropriate interventions addressing social determinants for attainment of health equity [94, 95]. There are 70,000 CHWs in South Africa, employed by over 3,000 NGOs [51]. The government has made efforts to formalise and integrate CHWs into the public system, but progress is slow, uneven, with low coverage and inadequate staffing [52, 96, 97]. The situation is not particular to South Africa. CHWs everywhere are denied fair pay, safe working conditions and basic recognition and value [98, 99]. It is unsurprising that while community mobilisation is recognised as a critical CHW competency, it is not effectively operationalised [100–102].

The PAR process addressed some of these issues. CHWs developed new capabilities for community mobilisation as well as new skills and confidence in complex analyses, public speaking, and reporting. Through regular engagement with higher levels of the system, their value, commitment, and competency were explicitly recognised. The authorities embraced the process; shared responsibility for health was welcomed in principle and pragmatically, and it was taken up locally [75].

Embedding the learning spaces into routine systems functions was critical. International evidence on participatory practices supports multiple inputs to support transformation, and that two-way interactions between ‘claimed’ (collective-controlled) and ‘invited’ (formal) spaces can be mutually empowering: informal spaces are flexible, inclusive, accessible, and responsive while formal management and planning are critical for service response [80]. We claimed and protected spaces for new forms of ‘everyday leadership’ rooted in community voice, cooperative learning and data and evidence, and worked with services to integrate these into routine functions [103–105]. In doing so, collective capabilities for community agency interacted with and influenced social and institutional structures. This supported the development of cooperative mind-sets, alliances, and new ways of thinking, increasing the visibility and legitimacy of the process.

Embedding in organisational contexts is thus necessary to support ‘learning health systems’ with locally relevant data and deliberation [106–109]. While local uptake was positive, transformative potential will require longer-term engagement, and with higher levels of the system. We identified multiple, overlapping complex social and institutional systems exerting influence over the spaces and processes in more and less visible ways [78]. Reconstituting spaces and connecting with those with the power to act will require focused efforts to maintain community representation and capacities, and to respond to influences exerted by organisational and social contexts that may limit the power to act [78, 110]. Complex systems perspectives encourage attention to these influences and dynamics to enhance community voice and participation in health [103, 111–113].

### Reflections and practical guidance: ‘radical potential with pitfalls’ [14]

The research team was based at or affiliated with the HDSS or Mpumalanga DoH, with some members based in the UK. Fieldwork was led by a researcher with clinical/research background, who resided in the setting. This conferred important insights and sensitivities relevant for the different groups with whom engagement was progressed. As the main collaborative environment, the HDSS was an important setting with a long-term presence in, and trust relationship with, rural communities and local health authorities. Located across many LMICs to support public systems, HDSSs are stable public health observatories occupying strategically important positions between health authorities and communities [114].

The research team navigated diverse worlds, engaging stakeholders with differing attitudes, opinions, education levels and understanding of community health priorities. The process relied on strong partnerships, relationships, and trust, which were nurtured throughout. Specific competencies and cultural sensitivities were necessary to build connections, communication, knowledge of community empowerment theory and practical strategies. Understanding of and sensitivity to cultural and social norms, and allowance for the time-intensive nature of the process were also necessary. Among the research team, there was a commitment to self-reflection and critique. Acknowledging that participation itself constitutes a form of power, participatory theory helped navigate pitfalls that “participation hides and maintains a set of power relations” [21] (pp. 11). As the process is taken up, risk of ‘dislocation from radical politics’ requires ongoing attention [14, 82].

With a critical view of participation, we worked to continually reflect, share, and progressively transfer power. We created supportive spaces enabling community voice, and subsequently dialogue and formal engagement with systems and services. There was prioritisation of relationships and inter-connections, mutual respect, dignity, and connectedness, and deliberate power-sharing. The roles of researchers to mediate these processes is therefore a further noteworthy element. In Thailand, for example, the National Health Assembly supports coalitions of civil society, government and academics in participatory health systems governance and is a prominent example of virtuous cycles of community voice and state/systems response [115].

The process was developed with the intention of providing a sustainable PHC component. Commitments to embed in the health system brought the process to facilities, where clinic outreach teams did not see it as imposed but adapted it as their own. The process showed potential to improve how services respond to community needs while identifying and managing power dynamics. The reiterative cycles allowed us to progressively advance expansive shifts as power building dynamics reflected internal capabilities followed by engaging with and insights into wider social and systems contexts. Using the EPF and LPF, we were able to document the processes of building and sustaining community power in rural PHC.

Power-building within and between cycles was markedly non-linear. And, as described above, institutional, social, and political contexts exerted considerable influences. Analyses of limiting power forces revealed intersecting biases. Our analysis reflects that, a deeply hierarchical system, and society, notwithstanding, nurturing and democratising the processes through which community agency interacts with and influences social and institutional structures can support shifts towards cooperative mind-sets, alliances, and new ways of thinking increasing the visibility and legitimacy of cooperation learning. Wider perspectives on power assert that it is fluid and circulates [21]. Future analyses should therefore focus on the ways that agency and structure combine, interact, and situate in contexts that institutionalise participation as a rights-based approach to health [15].

**Fig. 8:**
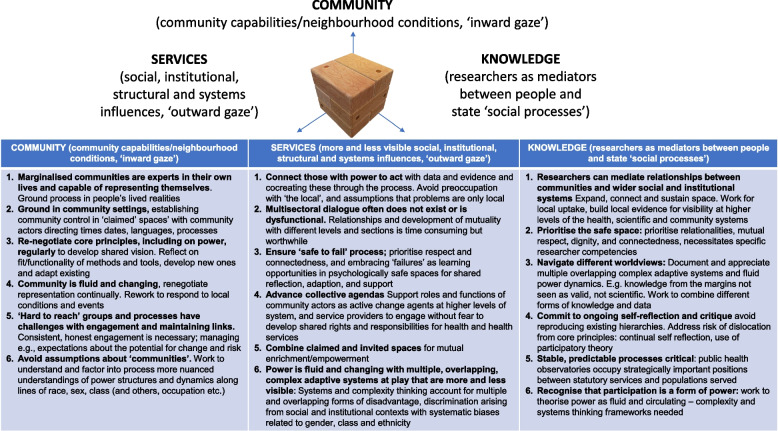
Practice framework to expand community power building as a sustainable component of PHC

In Fig. 8, we present framework to expand community power in PHC comprised on three pillars: (1) prioritising community capability-building, (2) building dialogue and trust in and navigating social and institutional contexts and (3) learning from the experiences of developing and sustaining authentic learning spaces. We previously used relational ideas to develop PAR methods focussed on mutual empowerment between service users and providers [68]. Building on this and combining insights and learning from the current analysis, we present the three pillars of the framework as a three-dimensional *space*, to depict collective capabilities being established, expanded, connected, and sustained in institutional and social contexts.

In terms of community capabilities (which we align to Popay’s ‘inward gaze’), the framework encourages attention to progressive expansion of community ownership and control, and regular revisiting of representation and voice. The importance of facilitation is also highlighted. We observed the necessity of sensitivities to, and cultural competencies around, working with so-called ‘hard-to-reach’ groups for more nuanced understandings of and responses to the needs and perspectives of otherwise excluded voices, and the power structures and dynamics within these. While these were key considerations to develop community capabilities, Popay’s work supports an appreciation that, in isolation, activities solely focused on ‘the inward gaze’ are insufficient for authentic and sustained community power-building.

In terms of supporting communities to work with other agencies and organisations (the ‘outward gaze’), there was a need to navigate multiple, complex, overlapping open systems each with inherent hierarchies and biases. Authentic participation required psychological safety to be a key characteristic of new multisectoral dialogue spaces. This supported shifts to more collective mindsets through which it was possible to find and advance collective agendas. This foundation enabled expansion as we connected with formal planning processes, blending ‘claimed’ and ‘invited’ spaces, which enabled us to understand how the process related to existing structures and systems and supported effectively working with those with some degree of power to act.

The framework also acknowledges the position and influence of researchers, and the importance of sustained, sensitive and assertive learning spaces. The research team were generally perceived as neutral arbiters with no allegiance other than realising synergies between services, science and society [116, 117]. Our role in creating ‘neutral safe spaces’ was important, as was a commitment valuing and combining diverse forms of knowledge, and the PAR framework encouraged continual reflection and critique. We regularly reflected, for example, on the tensions between a time-limited research project and sustainability objectives, and worked to leverage additional support to further develop and test the process with a continuous presence.

Figure 8 represents community power-building in a three-dimensional cube. In our process, the reiterative cycles amplified community power (effects) over time as the process embedded in context. The reiterative cycles supported shifts towards cooperative mind-sets, alliances, and new ways of thinking. This enabled expansive shifts as ‘power within’, ‘power with’, and ‘power to’ developed in each cycle, and overall. As described above, internal capabilities of community stakeholders were initially developed and embedded into formal structures and systems as we connected with the authorities. This, in turn, supported uptake; sustaining and embedding the process over space and time. Community power built when the process interacted with and influenced social and institutional structures, increasing visibility and legitimacy. And throughout, neutral safe spaces supported cooperative relationships and constructive dialogue. The power-building process should thus be considered *dynamically*: with appreciation of the process as expansive, as progressively sharing power, critically reflecting, and engaging with those with the power to act throughout. The framework is intended to encourage a critical appreciation of participation as deeply relational, non-linear, and gradual, prioritising mutuality and connectedness, and supporting ideas of power as fluid and circulating.

## Conclusion

Participation is widely accepted as an approach to support attainment of health equity. However, implementation remains a challenge and concerns remain about under-theorisation of power. In rural South Africa, despite structural revival of the DHS, there are few spaces for meaningful local engagement, learning and action. To address these gaps, we developed a theory-informed analysis of community power-building through multisectoral learning partnerships and developed practical guidance for the process as a sustainable PHC component. Marginalized community voices were engaged through structured processes to raise and frame issues of local importance. Power dynamics manifested through the co-creation, facilitation, and adaption of ‘safe spaces’ for dialogue and learning. Connecting community stakeholders with service providers, the reiterative cycles enabled new ways of thinking and collaborative mind-sets. The process needed time, space and a sensitive, inclusive, approach shifting power and control towards those most affected, and we worked with stakeholders to embed the process into routine PHC functions in the rural district. Despite challenges with implementation of collective action, impacts were observed in improved engagement, mutual understanding, and trust between service users and providers and new opportunities for learning. Impacts were also seen in demand for implementation outside the study setting.

Community power-building frameworks helped us account for how marginalized community voices were amplified. We identified and problematised local health priorities, while creating spaces for multi-stakeholder engagement with health and other sectors. On this basis, we progressed and reflected on solutions to address shared health concerns and how sustaining the process over space and time could be enabled and supported. Power frameworks also supported attention to systems and structural influences on community power-building. Adapting to changing circumstances and needs over time, we identified multiple, intersecting forces limiting community health action, especially by CHWs, related to race, gender, socio-economic status, and ethnicity. Nevertheless, COVID-19 underscored the need for real-time data-driven decision-making at local levels and through uptake by the system, new spaces where people can use evidence and make decisions were developed. Our findings illustrate the non-linearity and mutuality of power building, the challenges involved and the importance of balancing power while building spaces for capabilities to be developed and for engagement, especially with those with the power to act. We developed a view of participation as a form of power, as fluid and circulating, with radical potential and pitfalls. With a critical view of participation, attending to creating spaces to connect and mediate connections, sustaining, and expanding has the potential to develop new popular agency for community-based claims to health, though local action challenging wider structural conditions.

## Supplementary Information

**Additional file 1: Supplementary material 1.** Community-stakeholder workshops (n=16).

**Additional file 2: Supplementary material 2.** Workshops with communities and local authorities (n=6).

**Additional file 3: Supplementary material 3.** CHW community mobilization training manual.

**Additional file 4: Supplementary material 4.** CHW community mobilization training with rapid PAR workshops (n=15).

## Data Availability

The datasets generated and/or analysed during the study are not publicly available. Public deposition of the dataset would be in breach of the data management plan (DMP) within the study protocol approved by the research ethics boards in South Africa, the UK, and the permission for the study granted by the provincial health research committee, as well as the DMP within the funding proposal and the conditions upon which the research funding was granted. The data are, however, available from the corresponding author on reasonable request. A non-author point-of-contact (where authors are not available) is achds@abdn.ac.uk.

## Abbreviations

AOD: Alcohol and other drugs
CDF: Community Development Forum
CHW: Community Health Workers
COVID-19: Coronavirus Infectious Disease 2019
DoH: Department of Health
EPF: Emancipatory Power Framework
HDSS: Health and Socio-Demographic Surveillance System
HIV/AIDS: Human Immuno-Deficiency Syndrome/Acquired Immune Deficiency Syndrome
LMIC: Low- and middle-income countries
LPF: Limiting Power Framework
MRC: Medical Research Council
NGO: Non-governmental Organisation
NHI: National Health Insurance
OM: Operational Manager
OTL: Outreach team Leader
PAR: Participatory Action Research
PHC: Primary Health Care
TB: Tuberculosis
UHC: Universal Health Coverage
VA: Verbal Autopsy
VAPAR: Verbal Autopsy with Participatory Action Research
WBPHCOTs: Ward-based Primary Healthcare Outreach Teams
WHO: World Health Organization
