# Supplementary material for: Realising radical potential: building community power in primary health care through Participatory Action Research
Source: Int J Equity Health. 2023 May 17;22:94. doi: 10.1186/s12939-023-01894-7 (PMC10189714; doi:10.1186/s12939-023-01894-7)
Supplement: Supplementary file 1 — Additional file 1: Supplementary material 1. Community-stakeholder workshops (n=16). [file 12939_2023_1894_MOESM1_ESM.docx]

Supplementary material 1: Community-stakeholder workshops (n=16)

| Work-  shop* | Villages | Weekly topic | Tool/technique | Description |
| --- | --- | --- | --- | --- |
| 1-3 | A1  B1  C1 | Topic selection | Ranking, voting | Identify priority health topic of relevance to community. A list of health priorities developed during the discussions, after which participants voted for the topics of highest relevance. Voting progressed through rounds with discussion and agreement. |
| 4-6 | A1 and A2 B1 and B2 C1 and C2 | Problems and causes | Problem tree | Unpack/understand nominated topics from different perspectives. Through facilitated discussions using a tree diagram visible to all, participants identified cause-and-effect relationships at various levels from root (tree roots) to intermediary causes (trunk and branches) and consequences and other effects (tree pods) building subjective perspectives into shared accounts through consensus. |
| 7-9 | A1 and A2 B1 and B2 C1 and C2 | Actors and impacts | Venn diagrams | Understand impacts and actors. Collective account developed with Venn diagram of cardboard circles of different sizes and colours to indicate interactions between various actors and institutions, identifying internal and external organisations active in the topic and how they relate to one another in terms of contact and collaboration. |
| 10-12 | A1 and A2 B1 and B2 C1 and C2 | Action agendas | Action pathways | Articulate overall goal(s) to address issues identified and visualise and depict stepwise actions and actors to achieve these. Action pathway collectively developed to represent moving towards a desired goal via a series of interconnected events. |
| 13 | ABC | Problems and causes | Problem tree | As per workshop 2. |
| 14 | ABC | Actors and impacts | Venn diagrams | As per workshop 3. |
| 15 | ABC | Action agendas | Action pathways | As per workshop 4. |
| 16 | ABC | Reflections | Facilitated discussion | Reflect on experiences, outputs and how the process should be carried forward to engage government and non-governmental organisations. Participants discuss differences and similarities between the village-based group outputs through facilitated discussions, cross-verify each other’s outputs and reflect on the process and future development. |
| 4-16 | ABC | Lived experience | Photovoice | Basic training in photography, research ethics and digital cameras to take photographs to illustrate topic or condition via physical environments. Photographs presented and discussed in meetings and captions developed to describe what the image conveys. |

A1-C1: Original participants; A2-C2: New participants; ABC: 3 villages combined; ** All village-based discussion groups progressed through the sequence independently, coming together for workshops 13-16 to build further consensus, verify outputs, and reflect on process and next steps
