# Supplementary material for: Realising radical potential: building community power in primary health care through Participatory Action Research
Source: Int J Equity Health. 2023 May 17;22:94. doi: 10.1186/s12939-023-01894-7 (PMC10189714; doi:10.1186/s12939-023-01894-7)
Supplement: Supplementary file 2 — Additional file 2: Supplementary material 2. Workshops with communities and local authorities (n=6). [file 12939_2023_1894_MOESM2_ESM.docx]

Supplementary material 2: Workshops with communities and local authorities (n=6)

| Work-shop | Topic | Description |
| --- | --- | --- |
| 1 | Analyse 1 | VAPAR evidence presented to health system stakeholders (planners and managers) (n=12-16 representatives). Facilitated group work (a) group model building to define the focus and boundaries of the problem, formulate problem statements and consider potential strategies to address these; (b) pathways analysis to identify actions to address issues identified and (c) appraisal of actions in terms of affordability, acceptability, regulatory impact, fit with other policies, implement-ability, timescale, consequences and health and social impacts, with probabilities of success due to proposed changes estimated in light of evidence. Finally, a process for the remaining meetings discussed and agreed by consensus. |
| 2 | Analyse 1 | Develop recommendations and action plans with stakeholders including participants from within and outside the health system and with higher and lower-level planners and managers to foster a whole systems perspective. Here the panel was inclusive of participants from the stakeholder group, as well as those identified in Workshop 1. Workshop provided orientation to complex whole systems approaches, health equity, coproduction, and partnerships research. VAPAR evidence re-presented, and participants re-visited and further developed the group models and pathways analyses from prior workshop from an inter-sectoral perspective to develop whole system understandings of the complex inter-relationships of factors and enable identification of potential leverage points to influence systems performance in sectors within and beyond health. Participants also re-visited and re-appraised action plans and discuss and agree a process for the remaining workshop. |
| 3 | Analyse 1 | Verification and ratification of recommendations and action plans. Stakeholder reflections on, and adaptations to, the process, and adaptations made to facilitate stakeholder ownership and control. Representatives from prior community-led element discuss evidence, review findings, and consider sustainability in health systems through partnerships between practitioners, communities, and researchers. Codesign the subsequent ‘plan’ element in terms of overall objectives, timings, participants, and process |
| 4 | Plan 1, 2 | Re-orientation to VAPAR, health systems research, coproduction, and partnerships research. VAPAR evidence re-presented, and participants re-visit group models and pathways analyses from prior ‘analyse’ element from an inter-sectoral perspective to develop whole system understandings of the complex inter-relationships of factors and enable identification of potential leverage points to influence systems performance in sectors within and beyond health. Recommendations developed in the prior step conveyed and further interrogation and development of responses from a general systems perspective facilitated. Appraisal of actions in terms of affordability, acceptability, regulatory impact, fit with other policies, implement-ability, timescale, consequences and health and social impacts, with probabilities of success due to proposed changes estimated considering evidence. Finally, a process for the remaining meetings discussed and agreed by consensus. |
| 5 | Plan 3 | Final verification and ratification of appraised action plans. Stakeholder reflections on, and adaptations to, the process, and adaptations made to facilitate stakeholder ownership and control. Representatives from the community-led element of the cycle to discuss the evidence, review findings, and consider sustainability in health systems through partnerships between practitioners, communities, and researchers. The final workshop will also outline the subsequent element (implementation and monitoring) in terms of overall objectives, timings, participants, and processes. |
