# Supplementary material for: Realising radical potential: building community power in primary health care through Participatory Action Research
Source: Int J Equity Health. 2023 May 17;22:94. doi: 10.1186/s12939-023-01894-7 (PMC10189714; doi:10.1186/s12939-023-01894-7)
Supplement: Supplementary file 4 [file 12939_2023_1894_MOESM4_ESM.docx]

Supplementary material 4: CHW community mobilization training with rapid PAR workshops (n=15)

| Week/Workshop | Focus | Participants | Facilitator/s | Activities |
| --- | --- | --- | --- | --- |
| Week 1 Workshop 1 | Introduction Training | CHWs (9) Community mentors (6) Clinic nurses (3) Clinic committee (3) | Researchers | Training in PAR tool 1 (listing/ranking/rating) to support and orient CHWs and community mentors to identify and prioritize focus health issue(s). |
| Weeks 2-3 Workshop 2 | Training | CHWs (9) Community mentors (6) | Researchers | Training in PAR tool 2 (problem tree) to identify priority health related condition, facilitation and consensus building and community engagement - engaging active participant selection and representation (n=3 for each CHW). Training in PAR tool 3 (Photovoice) to convey lived experience visually. Basic photography training conveying topics in context and showcased in discourses. Captions on images. Baseline interviews. |
| Week 4 Workshop 3-5 | Problematising | CHWs (3) Community mentors (2) Community pax. (9) | CHWs and community mentors | Apply PAR tools 2 and 3 to understand priority health issue from different perspectives, identifying cause-and-effect relationships, consequences, and other effects, building shared accounts. Orient community pax. to collection of visual data. |
| Week 5 Workshop 6 | Training | CHWs (9) Community mentors (6) | Researchers | Reflect on week 4 workshops (3-5). Training in PAR tool 4 (Venn diagram) to identify actors and organizations involved with the topic and how they relate to one another with regards to collaboration and contact. |
| Week 6 Workshop 7-9 | Actors and impacts | CHWs (3) Community mentors (2) Community pax. (9) | CHWs and community mentors | Apply PAR tool 4 to understand actors and impacts. Collective account indicating relationships and interactions between various actors and institutions. |
| Week 7 Workshop 10 | Training | CHWs (9) Community mentors (6) | Researchers | Reflect on week 6 workshops (7-9). Training in PAR tool 5 (Action planning) to collectively articulate overall goal(s) and develop series of interconnected steps and events to achieve these. |
| Week 8 Workshop 11-13 | Action planning | CHWs (3) Community mentors (2) Community pax. (9) | CHWs and community mentors | Apply PAR tool 5 to agree overall goal(s) to address issues identified and develop stepwise actions to achieve these. Draw on problem tree and Venn diagram to collectively articulate a desired goal and a series of interconnected steps and events. |
| Week 9 Workshop 14 | Synthesis and process for engaging authorities | CHWs (9) Community mentors (6) Clinic nurses (3) Clinic committee (3) | Researchers | Reflect on week 8 workshops (11-13). Codesign/plan taking local action plans to multisectoral fora for analysis and planning. End-line interviews. |
| Week 10 Workshop 15 | Reflect on evidence, planning engagement | CHWs (9) Community mentors (6) Clinic nurses (3) and Clinic committee (3) Community pax. (27) | CHWs and community mentors | Present and finalise PAR data for 3 villages. End-line interviews. |

N.B.: Shaded rows indicate community workshops held in each of 3 villages; clear rows denote training/peer support workshops. ‘Pax.’ denotes participants.
